# “Something is wrong!” A qualitative study of racial disparities in parental experiences of OSA detection in their child

**DOI:** 10.3389/frsle.2023.1193539

**Published:** 2023-08-16

**Authors:** Alicia Chung, Leone Farquharson, Akila Gopalkrishnan, Sarah Morsbach Honaker

**Affiliations:** ^1^Department of Population Health, Center for Early Childhood Health and Development, NYU Grossman School of Medicine, New York, NY, United States; ^2^Every Horizon NGO, Toronto, ON, Canada; ^3^Child and Family Equity Prevention and Research Lab, The University of Southern Mississippi, Hattiesburg, MS, United States; ^4^Department of Pediatrics, Indiana University School of Medicine, Indianapolis, IN, United States

**Keywords:** obstructive sleep apnea, pediatric sleep, sleep disparities, parent experiences, race

## Abstract

**Introduction:**

Approximately 3% of American children are affected by obstructive sleep apnea (OSA), yet Black children are 2–4 times more likely to experience OSA compared to White children. Little is known about parental experiences in detection, diagnosis, and treatment of OSA in their child, and how these experiences may differ by race. The study objective was to highlight convergent and divergent experiences between and across Black and White parents in the OSA detection process for their child.

**Methods:**

We conducted 27 semi-structured interviews with mothers whose child was referred for a diagnostic overnight polysomnogram (PSG) to assess for OSA. Parents described how their child was referred for a PSG and their perceptions and feelings throughout the detection process. Data were analyzed using a thematic descriptive approach. Frequency of themes were examined by race. Themes that were unique to one racial group were categorized as divergent, whereas themes described by individuals from both groups were categorized as convergent. Within the convergent themes, we examined the prevalence within each racial group, noting those that were more prevalent (>10% difference in prevalence) in one race or the other.

**Results:**

The sample included 19 Black and 8 White mothers, who were 36 years old on average. Qualitative analysis yielded 21 themes across 5 categories that captured divergent and convergent experiences across Black and White mothers during the OSA detection process for their child. Divergent themes that were unique to Black mothers included *It Takes a Village—Teacher, Misplaced Blame, Missing the Day/night Connection, Trust in Provider*, and the belief that *Snoring is Normal*. Only one divergent theme among White parents emerged, worries about *Dying in Ones Sleep*. Additional convergent themes were identified that were more prevalent in one race compared to the other.

**Discussion:**

Black and White mothers experienced different paths to detection and diagnosis for their child's sleep disordered breathing, that are affected by individual awareness, education, patient-provider interactions, and experiences with the healthcare system. Divergent themes such as *Misplaced Blame* among Black mothers were a potential indication of racism and health disparities.

## 1. Introduction

Pediatric obstructive sleep APNEA (OSA) is a grave condition that may have lasting neurodevelopmental and health outcomes if left untreated (Stepanski et al., 1999; Huang and Guilleminault, 2017; Gouthro and Slowik, 2022). Poor academic performance, neurobehavioral impairment, and reduced quality of life are consistently associated with untreated OSA (Spilsbury et al., 2006; Strocker and Shapiro, 2007; Capdevila et al., 2008). Early diagnosis and treatment are crucial to circumvent poor child development outcomes. The path to OSA detection often varies; for example, the process can be initiated by parent concerns, provider screening, or as an incidental finding (Honaker et al., 2022). We posit that this path is influenced by parent experiences that are shaped by race.

While OSA affects up to 5% of American children, black children are 2–4 times more likely to experience OSA compared to white children (Levers-Landis and Redline, 2007; Paruthi, 2022). Evidence suggests that median time to diagnosis can be up to five times longer among black children compared to their white counterparts (Kilaikode et al., 2018). Delayed diagnosis may contribute to broader disparities in health and academic functioning.

The literature identifies race and socio-economic status (SES) as key risk factors associated with pediatric sleep disparities. The previous paragraph highlights the association between minority race and greater OSA risk/delayed detection (Stamatakis et al., 2007; Giddens et al., 2022). In addition, research shows that lower SES, as measured by income and education, is linked to sleep disparities and OSA risk over the life course, even when controlling for race (Spilsbury et al., 2006; Xie et al., 2018). For example, Spilsbury et al. (2006) found that primary caregiver education and other components of neighborhood disadvantage were associated with pediatric OSA severity and diagnosis, particularly among African–American children. Xie et al. (2018) identified single-parent households, public insurance, and proximity to care centers as factors affecting OSA health outcomes. The interaction between SES and race is likely to amplify the risk for OSA and delayed diagnosis in black children with lower SES. Family support is identified as a positive determinant of OSA patient outcomes and could be a key factor in the detection and treatment of OSA in disadvantaged populations (Luyster et al., 2016). It is important to consider both race and SES in addressing pediatric sleep disparities and improving detection and treatment of OSA, especially among historically disadvantaged black populations (Danielson, 2022; Park et al., 2022). Given that race is a social construct influenced by historical and contemporary manifestations of structural racism, it is important to examine how parents' race may affect their OSA detection journey experience (e.g., interactions with the education or healthcare system).

In our previous work, we described parental experiences with OSA detection in their child via qualitative analysis of interviews with a diverse sample of parents. For example, we found that parents had varying experiences during their OSA detection process in their children, which highlighted a need for dissemination of accurate OSA information targeting parents, as well as systemic strategies between medical providers, schools and parents that can lead to earlier detection (Honaker et al., 2022). Missing from the literature, however, are studies examining the role of race in parental experience with pediatric OSA detection. Thus, our objective was to examine shared and unique parental experiences with pediatric OSA detection in black and white parents.

## 2. Methods

### 2.1. Theoretical framework

We applied grounded theory as our methodological approach to guide qualitative analysis. Grounded theory is an evidence-based method for analyzing qualitative research that enables the generation of new understandings of a phenomenon to emerge from data that has been collected and organized in a systematic way (Noble and Mitchell, 2016). This theoretical approach was selected as it places the parent experiences at the center of the analysis, identifying themes driven by the data rather than through a-priori codes. This methodological approach is well-recognized in qualitative research and is described in greater detail below.

### 2.2. Participants

Participant interview data was selected from a larger sample described in detail elsewhere (Honaker et al., 2022). Briefly, all participants were parents of a child who had been referred to the Riley Sleep Disorders Center in Indianapolis, Indiana for a diagnostic sleep study (polysomnogram; PSG) for the indication of sleep-disordered breathing. Parents of Black children and/or children living in historically disinvested neighborhoods were over-recruited given their high-risk for OSA and under-detection rates. The sample size (*n* = 30) was not selected a-priori but interviews were conducted until thematic saturation was reached.

For the current analysis (*n* = 27), parents were included only if they identified as: (1) White non-Hispanic (*n* = 8), with no additional races or ethnicities endorsed, or (2) Black, with or without additional races or Hispanic ethnicity endorsed (*n* = 19). We classified parents who self-identified as Black and another race as Black, given that bi-racial or multiracial Black groups, tend to ascribe to Black identity and political views (Davenport, 2016), and that our focus on parent experiences with OSA detection shaped by race, would be reflected as such procedure.

### 2.3. Protocol

A research associate invited and consented eligible participants over the phone. During the 15-min phone call, demographic and OSA knowledge questions were administered, in addition to scheduling the subsequent phone call. Interviews were conducted via phone. The interviewer used a semi-structured interview guide (Appendix A) that incorporated questions about how children came to be referred for a PSG as well as parental experiences, perceptions, and feelings throughout the detection process. Interviews lasted ~30 min and participants received a $40 gift card. The study was approved by the Indiana University School of Medicine Institutional Review Board. Informed consent was obtained from all study participants during the initial phone call.

### 2.4. Socio-demographics

The parent of the target child referred for PSG reported their age, race/ethnicity, highest education level attained, and relationship to the child, as well as their child's age, race/ethnicity, and sex.

### 2.5. Analytic approach

A descriptive approach was used to characterize the sample. Qualitative analysis included thematic analysis of nodes, codes, and categories that emerged from the data. A study author reviewed the interview data for accuracy and uploaded the transcripts to NVivo Version 12 for analysis. We applied a two-phase approach as part of our collaborative node development, coding, and categorizing that involved all study authors during team meetings. A random sample of interviews (*n* = 5, 16.6%) were double-coded with a Cohen's Kappa of 0.81. Several strategies were employed to promote methodological rigor, such as clear documentation and description of analytic process, reporting the sample sizes for themes to provide context about the commonality of theme, assessing interrater reliability, and describing protocols for addressing coder disagreement (Johnson et al., 2020). Additional details on our analytic approach can be found in our previously published work with the larger sample (Honaker et al., 2022).

Themes were classified as divergent between Black and White parents if they were reported only by members of one racial/ethnic group and not the other. Divergent themes could be unique to Black parents or to White parents. Convergent themes were those experienced by members of both groups. To determine whether convergent themes were more prevalent in one group than the other, a cut-off of 10% difference between the White and Black parent groups was applied (Hebert and Howell, 2008). Convergent themes thus could be comparable between groups (i.e., occurring in both group at similar frequency), more prevalent in Black parents, or more prevalent in White parents.

## 3. Results

### 3.1. Sample descriptives

Among the 27 participants in the study sample, participants were primarily mothers (93%), who identified as Black (70%, *n* = 19) or White (30%, *n* = 8). Mothers were 36.8 (S.D. 10) years old on average. Black children were 8.9 years old on average, and White children were 7.5 years old on average. Fifty-six percent of the children were female. All mothers were enrolled in Medicaid. Participants resided in the metro setting of Indianapolis, Indiana. See Table 1 for participant sociodemographic characteristics.

**Table 1:** Participant sociodemographic characteristics.

**Characteristics**	**Participants (*****n*** = **27)**	
Age, mean (SD)	36.8 (10)	
Parent gender, female, *n* (%)	27 (100)	
**Race/ethnicity**, ***n*** **(%)**		
Black/African American non-Hispanic	19 (70)	
White non-Hispanic	8 (30)	
	**Black**	**White**
**Highest educational attainment**, ***n*** **(%)**		
Some high school	1 (6)	0
High school degree or GED	2 (12)	2 (12)
Some college	10 (59)	3 (18)
College degree	4 (24)	2 (12)
Child age, mean	8.9 (3)	7.5 (3)
Child gender, female, *n* (%)	15 (56)

To provide context for the analysis, rates of OSA diagnosis, PSG completion, and OSA treatment are presented by race. Parents interviewed were at a variety of stages in the OSA detection process (e.g., treatment completed; surgery pending; no treatment needed; PSG scheduled; decided not to complete PSG). These estimates are presented descriptively as the study was not powered to conduct statistical comparisons, nor was this consistent with the study objective. The rate of OSA diagnosis was 58% for Black children and 50% for White children. PSG completion rates were comparable for both Black and White children, 32% for Black, and 25% for White children, respectively. At the time of the interview, 16% of Black children and 38% of White children had received treatment for their OSA.

### 3.2. Overall themes

Twenty-one themes were classified into five categories (Table 2): *Variable Signs and Symptoms, PSG Facilitators and Barriers, OSA Knowledge, Healthcare Experiences*, and *Parent Experiences*. A description of the themes is included in Table 2. Additional details and examples of themes can be found in our previously published work with the larger sample (Honaker et al., 2022).

**Table 2:** Parental experience with pediatric OSA detection: categories, themes, and description.

**Category**		**Theme**	**Theme description**
Variable signs and symptoms		Day and night	Parent observation of both daytime symptoms (e.g., sleepiness, hyperactivity) and nighttime symptoms (e.g., snoring) in their child.
		Something is wrong	Parent perception that their child had a problem that needed to be addressed.
		Missing the night-day connection	Even when daytime and nighttime symptoms were noticed, parents may not have perceived these as associated until much later in the detection process.
		It takes a village	Many different people in the wider community played a role in initiating the OSA detection process.
		Misplaced blame	Unfair blame attributed to the parent or child due to the child's OSA.
Facilitators and barriers to PSG	Facilitators	Wanting to know	Perception of the PSG as providing valuable health information, including in cases where the PSG was negative.
		Trust in provider	Parent trust in the provider who made the PSG referral was a facilitator to PSG completion.
	Barriers	Institutional follow-up	Parents experienced PSG delays (e.g., scheduling delays, not getting a report) on the part of the healthcare institution.
		Is it really needed?	Parents reported uncertainty about the necessity or priority of the PSG for their child's care.
		Structural barriers	Structural barriers to PSG completion such as competing responsibilities and priorities or cost.
OSA knowledge	Misinformation	Snoring is normal	Parent perception of their child's snoring as normal, prior to OSA detection
	Missing information	Cardiovascular consequences	Lack of awareness regarding the connection between untreated OSA and cardiovascular risk.
	Sources of knowledge	Own experience	Parental knowledge of OSA from personal experience or from family members.
		Health care provider	Healthcare providers (e.g., PCPs, specialists and PSG technicians) as sources of OSA knowledge for parents and their child.
Healthcare experience		Low threshold for raising concerns	Minimal hesitation or reservation about raising concerns with their child's PCP.
		Parent as advocate	Parent advocating for the child within the healthcare system in order to overcome a barrier or delay in the process.
		Institutional delays	Delays in the detection process resulting from lack of care coordination
		Lingering questions and concerns	Lingering questions/concerns about their child's wellness after the OSA detection had concluded.
Parent experiences		Worries	Expressions of worry based on the potential short-and long-term consequences of poor breathing at night.
		Whatever it takes	The willingness to take any necessary steps to promote their child's wellbeing.
		Benefits of OSA detection and treatment	After OSA treatment, the perception of improvement in their child's functioning.

**Figure 1:**
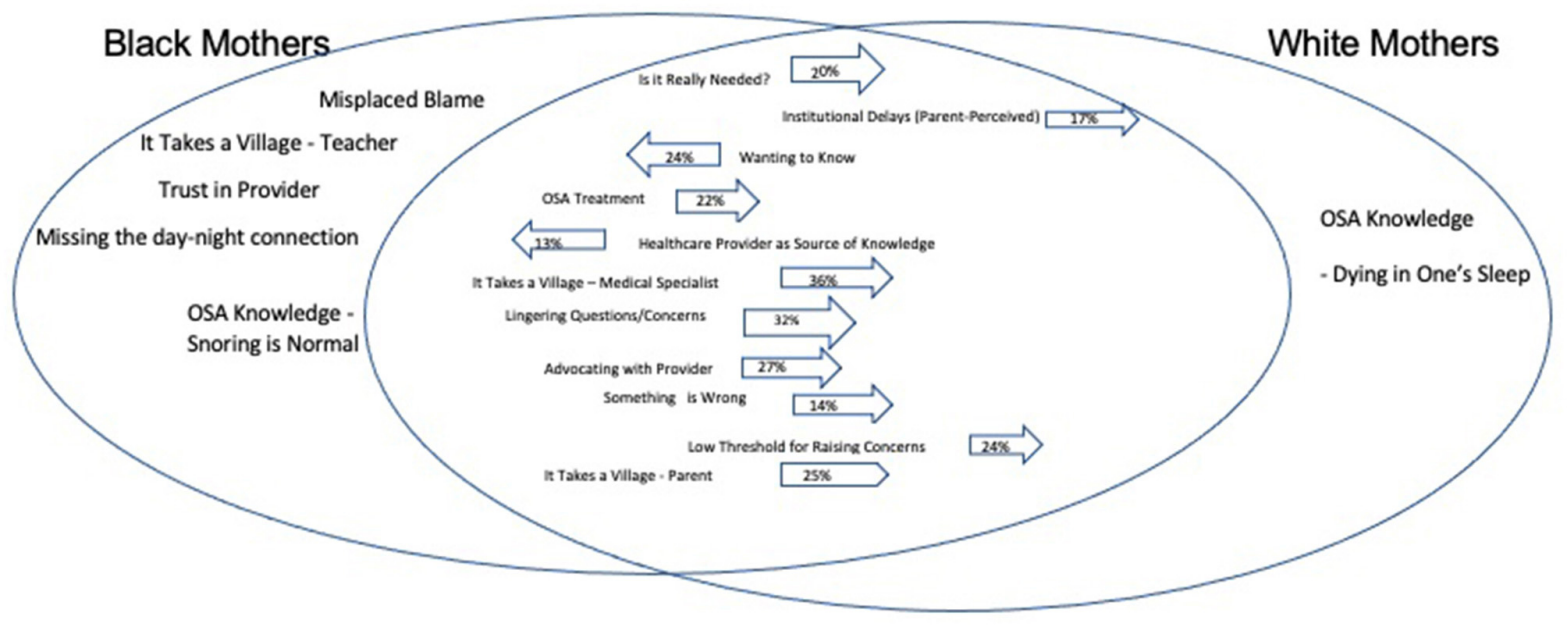
Venn diagram of the convergent and divergent parent experience with OSA diagnosis for their child by race. Convergent themes (reflecting those experienced by both Black and White mothers) are presented in the middle overlapping section of the two circles in the diagram. Convergent themes without an arrow were less experienced by both groups at comparable rates. Convergent themes with an arrow indicate a ≥10% difference in prevalence between Black (arrow pointing left) and White parents (arrow pointing right). Divergent themes (uniquely experienced by one racial group but not the other) are in the non-overlapping portions of the circles.

Convergent themes were reported in both Black and White mothers. Divergent themes were only reported by one race of mothers. Table 3 illustrates select quotes by Black and White mothers, organized by theme. Classification of themes as divergent or convergent are presented in Figure 1. Figure 1 is a Venn diagram of the convergent and divergent themes identified by mothers. This figure visually depicts themes that are overlapping experiences among all mothers, or distinct to each group.

**Table 3:** Select quotes of parent experiences with OSA detection by theme.

**Themes**	**Type of theme**	**Mothers' race**	**Select quote**
Misplaced blame (parent)	Divergent	Black	I'm like okay, I kind of know why he's sleeping [in school]. You all are saying it like I just don't put him to bed on time or something. *ID3*
		Black	She's not being rebellious. It's a medical issue. *ID2*
Is it really needed?	Convergent	Black	So, since, after I saw that things are not as bad as they were, I kind of relaxed. *ID21*
Whatever it takes	Convergent	Black	I just basically was like okay whatever we need to do to kind of get him to sleep better, I down for it. *ID8*
		White	And I'm like, I gotta hurry up and do something. *ID14*
Trust in the provider	Divergent	Black	He's a good, good doctor, I love him a lot. I love him a lot, because any time, in any concern that I've got, he helped me a lot. *ID9*
Wanting to know	Convergent	Black	I wanted to find out what it was, and I wanted to help her… *ID14*
		Black	I wanted to know what was going on. *ID22*
Institutional follow-up	Convergent	White	It started when my son was little and nobody did nothing about it. *ID18*
		White	Until I followed up with them, I would have never heard anything. *ID23*
Something is wrong	Convergent	Black	So now we are trying to figure out what's really going on, because now we know there's problems. *ID1*
		Black	I just knew that it was not, it wasn't supposed to be like that. *ID14*
Parent as advocate	Convergent	Black	[PCP] keep saying maybe she having some maybe cold allergy or something. I told them no, this is no cold allergy. *ID16*
		White	The patient has to be a little more proactive and responsible for taking care of [healthcare system's] job. *ID23*
Own experience as source of knowledge	Convergent	Black	I was really familiar with it because I had one before. *ID3*
Normalize snoring	Divergent	Black	I thought her snoring was, I thought it was normal, because all of us basically snore in my house. My other two children both snore. *ID26*
		Black	I thought she was just snoring. Most people snore, so I just thought she was snoring, that's normal. *ID1*
Healthcare provider as source of knowledge	Convergent	Black	[The PCP] said that it would be an overnight sleep, that she would be hooked up to the little machines and everything for them to monitor her sleep. *ID2*
Missing the day-night connection (daytime/nighttime symptoms)	Divergent	Black	In fact, we thought it's maybe, my mom was thinking maybe it's ADHD or something. *ID21*
		Black	That's where her bad attitude comes from, sleeping. She's always got a bad attitude, in a bad mood, so that's where the problem is, she's sleepy. *ID2*
Benefits of OSA detection and treatment	Convergent	White	From that day to now, I see him and he's doing great. *ID9*
		Black	She wakes up, because like on the weekends, before she had sleep apnea, she could sleep until you woke her up. Now, she will get up. *ID1*
Lingering questions and concerns	Convergent	Black	I don't know if she grew out of [snoring] though. I don't know what happened. *ID17*
Low threshold for raising concerns	Convergent	White	Any time I find an issue with any of my kids, I go straight to their PCP. *ID10*
		Black	If I have a concern about anything I feel like if a situation can't get better unless you address it. *ID8*
Cardiovascular consequences	Convergent	Black	I didn't learn about …the high blood pressure though, that is new. *ID8*
Worries	Convergent	White	He made me scared because I was what? What was that? He said like hmmm, hmmm. *ID9*
		Black	Actually, I was a little nervous because at times I did, I kind of caught him stop breathing. *ID4*
Structural barriers	Convergent	White	I was just too busy with work and my daughter and everything to do that, because we'd have to do it overnight. *ID27*
Institutional delays (parent-perceived)	Convergent	White	They kept saying that they kept calling to schedule an appointment but I never received a phone call. (ID12) Until I followed up with them, I would have never heard anything. (ID23)
It takes a village	Convergent	White	I had brought it up to her pediatrician who referred us to have a sleep study. (ID10)
		White	[The neurologist] said she's not having seizures, so that's fine. Then she said why don't we do the sleep study? (ID26)
		Black	I wanted to figure out what was going on with him…because the school kept saying he's sleeping at school. (ID3)

### 3.3. Divergent themes

Six themes classified as divergent themes unique to Black parents included *Misplaced Blame, Missing the Day/Night Connection, It Takes a Village—Teacher, Trust in Provider*, and *Snoring is Normal*. One identified theme that was unique to White parents, namely *OSA Knowledge—Dying in Ones Sleep*.

### 3.4. Convergent themes with comparable prevalence between groups

Five themes were convergent, with comparable prevalence between Black and White parents, including: *Daytime and Nighttime Symptoms, Worries, Whatever It Takes, It Takes. A Village—Primary Care Provider, and Lack of Knowledge (Cardiovascular Consequences)*.

### 3.5. Convergent themes more prominent among black mothers

Two themes that were present among both racial groups were more prominent among Black mothers compared to White mothers, specifically *Wanting to Know* (74 vs. 50%) as a facilitator for completing the PSG, and *Healthcare Provider as Source of Knowledge* (63 vs. 50%).

### 3.6. Convergent themes more prominent among white mothers

Themes more prevalent in White mothers than in Black mothers included *It Takes a Village (Parent;* 88 vs. 63%); *It Takes a Village* (*Medical Specialist;* 63 vs. 26%), *Something is Wrong* (88 vs. 74%), *Institutional Follow-up* (38 vs. 16%), *Advocating with Provider* (38 vs. 11%), *Low Threshold for Raising Concerns* (88 vs. 63%), *Institutional Delays (Parent-Perceived;* 38 vs. 21%), *Is it Really Needed?* (25 vs. 5%), and *Lingering Questions and Concerns* (38 vs. 5%).

## 4. Discussion

To our knowledge, this is the first qualitative study to examine parental experiences with pediatric OSA detection by race. Racial differences were identified in the path to child OSA diagnosis. As all parents in the study had children with Medicaid insurance, differences were likely driven primarily by racial factors and not socioeconomic status. Negative parent experiences due to bias or interactions with the school system may be an indication of racism and health disparities. For example, *Misplaced Blame* was a divergent theme only reported by Black mothers. One Black mother described her perception of being blamed for her child's sleepiness “The school was always—when they brought him home, they were always telling me he was sleeping at school. I'm like okay, I kind of know why he's sleeping. You all are saying it like I just don't put him to bed on time or something.” *(ID3)*. Similarly, another Black mother stated, “She would fall asleep more than once, and so I had told the teacher, I said she goes to bed on time, at a decent time*.” (ID1)*. The lack of OSA awareness among both the teacher and mother may be at play during early stages of the detection process. The negative parent-teacher interactions associated with parent blame may feel more pronounced among a minoritized population.

The divergent themes, “*It Takes a Village—Teacher*, an*d Trust in Provider*,” illustrate the influential role of each adult in a child's life on the pathway to OSA detection, particularly for Black children. There is a critical need for teacher OSA awareness and understanding, that undiagnosed OSA may manifest as behavioral problems in the classroom. This gap in teacher understanding, and potential implicit bias, may set Black children on a negative academic trajectory (Chin et al., 2020). Undiagnosed sleep apnea may contribute to poor academic performance or learning challenges (Galland et al., 2015). These learning challenges, coupled with race, may hamper student achievement for Black children, given that teacher observations about child sleep health in the classroom may be a contributing factor to a child's academic trajectory (Ursache et al., 2021). In addition, Black mothers are likely to experience discrimination in the education system, while exhibiting agency in their parent advocacy for their child (Rall and Holman, 2021).

Teachers and providers are influential in facilitating parents to obtain a PSG for their child and educating parents along the way during the process. The themes *Trust in Provider* and *Healthcare Provider as a Source of Knowledge* were unique to, or more prevalent in, Black children. Additionally, the convergent theme of *Healthcare Provider as a Source of Knowledge*, more prominent among Black mothers than White mothers, reinforces the need to examine potential provider bias. Provider bias has been associated with negative attitudes toward Black people, that could lead to poor health outcomes for their child (Hall et al., 2015). In addition, parent's source of OSA knowledge may be most affected by their own childhood experiences or education from a provider. Healthcare providers may be a trusted source of health information among some Black adults (Swoboda et al., 2018), similar to the mothers in our study. Yet, establishing interpersonal trust in the patient-provider relationship may depend heavily on the degree of communication, technical competence, perceived quest for profit, and potential racism perceived by a Black patient (Jacobs et al., 2006). Providers caring for Black patients should examine their racial consciousness and consider how race may affect their interpersonal relationship.

While treatment guidelines recommend universal OSA screening (Marcus et al., 2012), in many cases the detection pathway is initiated by parents raising concerns to their child's provider. Considering themes related to parental knowledge about OSA, both Black and White mothers reported OSA knowledge gaps that may have delayed their child's path to detection. For example, *Snoring is Normal and Missing the day-night Connection*, were divergent themes among Black mothers. Normalized snoring and not connecting daytime and nighttime symptoms suggest a knowledge gap in Black mothers that could delay OSA detection. Similarly, *OSA Knowledge—Dying in One's Sleep* was a divergent theme among White mothers, indicating a clear lack of understanding of OSA consequences.

The clinical significance of this study is its potential to contribute to earlier screening, detection and treatment of obstructive sleep apnea, especially among minoritized Black children. Earlier detection rates hold the clinical significance for potentially earlier rates of diagnosis and treatment that could affect child development outcomes, and potentially reduce health disparity outcomes. Delayed detection and diagnoses of OSA among Black children and adults (Dudley and Patel, 2016), may contribute to higher severity of disease state, and poor child development outcomes. The experiences of families in our study are consistent with this disparity, as Black children were about 2 years older than White children on average at the time of diagnosis. This OSA disparity may be due to a combination of lack of awareness, knowledge gaps, and miscommunication by the healthcare system. For instance, one Black mother stated, “I just wish the primary care had gotten back with me. I'm not certain as to who was supposed to let me know. Like the ENT called and told me about the breathing part, but she was like that's all we know*.” (ID7)* Timeliness from detection to diagnosis, is of paramount importance given the developmental affect untreated OSA may have on a child's physical health and development. Additionally, there also may have been differences in how mothers interpreted and acted upon observed OSA symptoms. A convergent theme more prominent among White mothers was a perception that, *Something is Wrong*, and *Is it Really Needed?* These themes fall in contrast to each other, as one serves as a facilitator to detection, while the other one is a barrier. Black mothers were more likely to express, *Wanting to Know* what was wrong with their child as a PSG facilitator. Other convergent experiences around *Worries*, and *Whatever It Takes*, were reported among mothers from both racial groups. Black mothers were more likely to describe positive experiences about the *Benefits of OSA Detection and Treatmen*t,” highlighting the importance of timely detection in Black children.

### 4.1. Limitations

Study limitations include the small sample size. In addition, generalizability is limited, given that study findings may only extend to the geographic and racial population similar to the sample in this study (e.g., 73% Black, living in Indianapolis, Indiana). Similarly, all participants were enrolled in Medicaid, thus socio-economic diversity was lacking. Further, measures of socio-economic diversity were limited to insurance type and maternal education. Finally, the experiences of parents of children older than 12-years-old were not reflected in this study.

### 4.2. Future directions

Each parent has a unique experience with OSA detection and diagnosis for their child. One Black mother commented, “She breathes easily and easier now and its more consistent breathing pattern and it made all the difference.” (*ID14)* A parent's journey to OSA diagnosis and treatment for their child may be taxing, but the resolution may bring hope. Future research should further investigate the impact of race on parental experiences related to pediatric OSA detection and diagnosis, promote validation of screening instruments in non-White groups, and evaluate strategies to reduce the barriers to diagnosis and optimal treatment among minoritized children. Additionally, OSA detection strategies should be initiated during infancy and early childhood to avoid potential delays in detection. Further implications for the field include the need for educational programs in medical schools and other post-graduate health curricula to prevent the under-diagnosis of pediatric OSA. Also, minority-specific interventions and public health campaigns targeting high-risk families are needed to bridge the gap in knowledge and cultural behaviors. Further research can address these gaps in research, and promote provider, educator, and parent awareness and knowledge of OSA symptoms, and how they may present themselves in a child of color, to ameliorate disparities and support better parent experiences.

## 5. Conclusion

There are both commonalities and differences on the OSA detection pathway for Black and White children and their parents. Differences in parent experiences are affected by individual awareness, education, patient-provider interactions, and experience with the school system that reflect bias. Parents and other adult figures in a child's life, such as school teacher or pediatrician, need targeted awareness of OSA and its behavioral and developmental consequences. Findings from this study are being applied toward the development of a health communication message to raise parental awareness about the signs and symptoms of OSA. In addition to raising awareness of OSA, structural changes are needed in the healthcare system to improve responsiveness and engagement with high-risk communities to reduce the effect of pediatric OSA disparities.

## Data availability statement

The original contributions presented in the study are included in the article/Supplementary material, further inquiries can be directed to the corresponding author/s.

## Ethics statement

The study was approved by the Indiana University School of Medicine Institutional Review Board. Written informed consent for participation was not required for this study in accordance with the national legislation and the institutional requirements.

## Author contributions

SH provided substantial contributions to the conception, design, data analysis, and interpretation of data. AG contributed to the data analysis and interpretation of data. LF revised and edited the manuscript critically for intellectual content. AC drafted the work and contributed substantially to the manuscript conception, design, data analysis, and interpretation of data. All authors contributed to the article and approved the submitted version.
