# Supplementary material for: “Something is wrong!” A qualitative study of racial disparities in parental experiences of OSA detection in their child
Source: Front Sleep. 2023 Aug 16;2:1193539. doi: 10.3389/frsle.2023.1193539 (PMC12713897; doi:10.3389/frsle.2023.1193539)
Supplement: Supplementary file 1 [file Table_1.DOCX]

Appendix A: Interview Guide

| **DETECTION EXPERIENCE** |
| --- |
| **Tell me the story of how your child was referred for a sleep study, starting from when you first noticed a sleep problem, to now.**  Optional Prompts:  *Tell me more about that. What happened next? How did you feel about that? What was your reaction to that?*  For parents whose child did not complete PSG, if this is not covered in the reply above, use the following prompt:  **Your child was referred for a sleep study appointment and has not had it done yet. Tell me what happened.**  **In thinking about the journey from when you first noticed a sleep problem to now, is there anything you wish had happened differently?** |
| **ACTIVATION** |
| **Sometimes when parents have a concern or question about their child, they choose to talk with their child’s doctor about it. How do you decide when to talk to your child’s doctor about a question or concern?**  **Optional Prompts:**  *Tell me about a time that you either talked to your child’s health care provider about a question or concern? How did it go? What made you decide to talk to the doctor about this?*  *Tell me about a time that you had a question or concern about your child’s health and did not talk to your child’s health care provider. What made this time different from the time above?* |
| **OSA KNOWLEDGE / PERCEPTIONS** |
| **Next, I am going to tell you some information about sleep apnea. I would like to know if there is anything that I say that you did not know, or that is surprising to you.**  **Obstructive sleep apnea is a sleep disorder that is found in about 2% of children. Children with sleep apnea have a problem with their airway, which is the passage between their mouth or nose and their lungs. This passage may close for several seconds while children are sleeping, so that no oxygen or not enough oxygen is entering the body. The child’s brain notices that there is not enough oxygen, causing the child to wake up. Often the child will return to sleep quickly. This may happen many times during the night, causing two problems. First, the child is waking all through the night and is not getting good sleep. Second, the child’s brain is not getting the oxygen it needs. Obstructive sleep apnea is not dangerous in the short term; children do not simply stop breathing and die from sleep apnea. However, obstructive sleep apnea can cause many problems for children over time. Many things can cause the child’s airway to close during sleep. Some common causes include having large tonsils, being overweight, or just the way a child’s face is shaped.**  **Is there anything I just told you that is surprising to you?**  **Snoring is the main sign of sleep apnea. Other signs might include snorting, gasping, or pauses in breathing. Some children with sleep apnea will seem sleepy during the day. However, sleep apnea can also cause other problems for children, such as hyperactivity, problems paying attention and learning, irritability, or other mood or behavior problems. Sleep apnea may also cause health problems such as high blood pressure or diabetes.**  **Is there anything I just told you that is surprising to you?**  **In children, the most common treatment for sleep apnea is surgery. Often, removing the child’s tonsils in their throat and removing their adenoids in their nose will open up the airway enough so that the child no longer has sleep apnea. A small percentage of children will still have sleep apnea after surgery, and will need to wear a pressure mask at night to keep their airway open.**  **Is there anything I just told you that is surprising to you?** |
